# Ammonia-oxidizing archaea adapted better to the dark, alkaline oligotrophic karst cave than their bacterial counterparts

**DOI:** 10.3389/fmicb.2024.1377721

**Published:** 2024-04-10

**Authors:** Qing Li, Xiaoyu Cheng, Xiaoyan Liu, Pengfei Gao, Hongmei Wang, Chuntian Su, Qibo Huang

**Affiliations:** ^1^School of Environmental Studies, China University of Geosciences, Wuhan, China; ^2^State Key Laboratory of Biogeology and Environmental Geology, China University of Geosciences, Wuhan, China; ^3^Institute of Karst Geology, CAGS/Key Laboratory of Karst Dynamics, MNR & GZAR, Guilin, China; ^4^Pingguo Guangxi, Karst Ecosystem, National Observation and Research Station, Pingguo, Guangxi, China

**Keywords:** ammonia-oxidizing community, nitrification, karst cave, niche breadth, subsurface biosphere

## Abstract

Subsurface karst caves provide unique opportunities to study the deep biosphere, shedding light on microbial contribution to elemental cycling. Although ammonia oxidation driven by both ammonia-oxidizing bacteria (AOB) and ammonia-oxidizing archaea (AOA) is well explored in soil and marine environments, our understanding in the subsurface biosphere still remained limited to date. To address this gap, weathered rock and sediment samples were collected from the Xincuntun Cave in Guilin City, an alkaline karst cave, and subjected to high-throughput sequencing and quantification of bacterial and archaeal *amoA*, along with determination of the potential nitrification rates (PNR). Results revealed that AOA dominated in ammonia oxidation, contributing 48–100% to the PNR, and AOA *amoA* gene copies outnumbered AOB by 2 to 6 orders. *Nitrososphaera* dominated in AOA communities, while *Nitrosopira* dominated AOB communities. AOA demonstrated significantly larger niche breadth than AOB. The development of AOA communities was influenced by deterministic processes (50.71%), while AOB communities were predominantly influenced by stochastic processes. TOC, NH_4_^+^, and Cl^−^ played crucial roles in shaping the compositions of ammonia oxidizers at the OTU level. Cross-domain co-occurrence networks highlighted the dominance of AOA nodes in the networks and positive associations between AOA and AOB, especially in the inner zone, suggesting collaborative effort to thrive in extreme environments. Their high gene copies, dominance in the interaction with ammonia oxidizing bacteria, expansive niche breadth and substantial contribution to PNR collectively confirmed that AOA better adapted to alkaline, oligotrophic karst caves environments, and thus play a fundamental role in nitrogen cycling in subsurface biosphere.

## 1 Introduction

Earth’s subsurface environments are isolated from phototrophic energy sources, which are characterized by oligotrophic condition and limitation in electron donors or electron acceptors (Jones et al., 2018). Microorganisms living in such environments are highly dependent on the oxidation of limited inorganic matter for energy (Zhang et al., 2018; Dong et al., 2020; Jones and Northup, 2021). Nevertheless, a large number of microbial cells is estimated to be 2 × 10^29^–6 × 10^29^ in the terrestrial subsurface biosphere excluding those in soils (Magnabosco et al., 2018). The subsurface biosphere serves as a fascinating place to decipher microbial dark matter and offer new knowledge about life, particularly in examining minimum energetic requirements and adaptations to oligotrophic environments.

Karst caves are known as subsurface extreme ecosystems with twilight or dark conditions, nutrient deprivation and isolation from the surficial environments, which harbor numerous chemoautotrophic microorganisms (Marques et al., 2019). Currently, progresses have been made on microbial communities, niche differentiation, community assembly and their correlation with environmental variables (Yun et al., 2016; Zhao et al., 2017; Cheng et al., 2019, 2021, 2022; Cao et al., 2021). Bacteria, microalgae, and fungi are found to be involved in the elemental cycles of carbon, nitrogen, sulfur, manganese, and iron, as well as in the dissolution and precipitation of limestone (Grobbelaar, 2000; Mulec, 2008; Sebela et al., 2015; Jones and Northup, 2021). Moreover, microbial functional groups such as ammonia-oxidizers are also been investigated and ammonia-oxidizing archaea (AOA) contribute more to ammonia oxidation than their bacterial counterparts as indicated by clone libraries in cave sediments (Zhao et al., 2017). Nevertheless, the limitation of the first-generation sequencing technique may not reveal the nitrifying communities comprehensively. Moreover, the adaption and ubiquitous occurrence of ammonia oxidizers in different cave habitats such as sediments and weathered rocks and how AOA and AOB interact with each other remain largely unexplored in the subsurface biosphere.

Nitrification is one of the fundamental biogeochemical processes mediated by microorganisms in natural environments, which has been well-reported in ecosystems such as soils, wetlands, farmland, estuary, and marine environments (He et al., 2007; Beman et al., 2012; Veresoglou et al., 2012; Phillips et al., 2015; Chen et al., 2019; Luvizott et al., 2019; Wei et al., 2023). The oxidation of ammonia (NH_3_) to nitrite (NO_2_^−^) is the first and rate-limiting step in nitrification, catalyzed by ammonia monooxygenase enzymes encoded by the *amoA* gene of AOA and AOB (Daims et al., 2015). Therefore, the *amoA* gene is widely used to explore ammonia-oxidizing communities in natural environments (Heiss et al., 2022). AOA and AOB are widely distributed in natural ecosystems, and they dominate ammonia-oxidizing communities under different conditions. Usually, AOA dominate in strong acidic environments with low ammonia concentration (Zhang et al., 2012; Kozlowski et al., 2016), whereas AOB is dominant in nitrogen rich environments (Di et al., 2009) due to their different substrate affinity (Martens-Habbena et al., 2009). Nevertheless, recent study showed that not all AOA possess such higher substrate affinity than AOB. In fact, the substrate affinity of ammonia-oxidizers correlated with their cell surface area to volume ratio (Jung et al., 2022). These new information on enzyme kinetic may indicate more complexity about niche differentiation between AOA and AOB. In addition, oxygen concentration, light condition, temperature, metal and organic compound also contribute to their distinct ecological niche differentiation in natural environments (Liu et al., 2015; Ouyang et al., 2017; Cheng et al., 2019; Khanom et al., 2021). The isolation and relative stable conditions create specific microhabitats within caves, especially loose sediments on the ground and weathered rocks of the cave passage (Cheng et al., 2023; Liu X. Y. et al., 2023), providing excellent conditions for the exploration of the niche differences of ammonia-oxidizing microorganisms in oligotrophic environments. However, the distribution of AOA and AOB and the ecological processes responsible for their development in these unique microhabitats remain poorly understood.

The theory of microbial assembly based on ecological niches is one of the universal tools to study microbial communities (Stegen et al., 2012; Ferrenberg et al., 2016; Yuan et al., 2019; Zhao et al., 2019a; Yu et al., 2021; Wang et al., 2024). Deterministic processes are the selection and filtering of species by ecological choices imposed by biotic and abiotic factors, while stochastic processes are the role of unpredictable interventions such as births and deaths on microbial communities (Zhou and Ning, 2017; Zhang et al., 2022; Fang W. K. et al., 2023). Ecological guilds exhibit different community structures due to different responses to environmental selection (Zhao et al., 2019b). Studies have clearly demonstrated the strong niche specificity of bacterial communities in loose sediments and those living on weathered rocks (Cao et al., 2021; Liu X. Y. et al., 2023) as well as for methanotrophs (Cheng et al., 2022), which may be also true in microbial functional groups involved in nitrification. If it is the case, what ecological processes contribute to the differences in ammonia-oxidizing communities in different niches in karst caves?

To fill these knowledge gaps, we collected loose sediment and weathered rock samples from the Xincuntun Cave in Guilin city, along the cave passage and subjected to amplicon high-throughput sequencing and quantification of the *amoA* gene and PNR (potential nitrification rate) measurement. Our aims are to investigate: (i) composition and niche differentiation of ammonia-oxidizing microbial communities; (ii) potential role of the environmental factors in ecological niche differentiation; (iii) the contribution of AOA and AOB to nitrification and their adaption to subsurface caves.

## 2 Materials and methods

### 2.1 Site description

The Xincuntun (XCT) Cave is a pristine karst cave without any tourists, located in Yongfu County, Guilin city, Guangxi Province (24°58′38.5″N, 109°44′15.7″E). It has a subtropical monsoon climate with an annual average temperature of 18.8°C and an annual average rainfall of 1,950 mm, which is mainly concentrated in March to August. The XCT Cave consists of two branches with a total length of 386 m. We sampled the left branch in this study, which is 100 m long, 2–7 m high, and about 3 m wide.

### 2.2 Sample collection

The weathered rock (W) and loose sediment (S) samples were collected from the cave with an interval of 20 m. The first two sampling sites were located in the entrance nearby zone with weak light (designated as ENZ), whereas the other three sampling sites located in the dark inner zones (IZ). Surface sediment samples were collected by five-point sampling method with a depth <5 cm with sterilized shovels and weathered rock samples were gently scraped from the weathered cave wall. All samples were stored in 50 mL sterile centrifuge tubes and kept on ice. They were immediately stored in freezer upon arrival in the hotel (−20°C). All utensils used for sampling were sterilized beforehand, and sterile gloves and masks were worn throughout the sampling to avoid contamination. Samples were transported back to the laboratory on ice in an insulated box and stored at −80°C for future use.

### 2.3 Physicochemical analysis parameters

Samples were freeze-dried for 24 h using a freeze-dryer (ALPHA 1-2LD, Christ, Germany). 10 g of ground sample was mixed with ultrapure water in a ratio of 1:5 (w/v), shaken for 5 min (Vortex-Genie^®^2, QIAGEN, Germany) and centrifuged at 6,800 × *g* (TGL-16A, Changsha) for 15 min. The supernatant was filtered through a 0.22 μm membrane, acidified with 3 M HCl for cation except NH_4_^+^ measurement using ICS-600 (Thermo, United States). The ammonium of the filtrate without acidification was measured by the salicylic acid assay (Kandeler and Gerber, 1988), and the filtrate without acidification was determined for anion with ICP-OES (iCAP 7,600+) ion chromatography. pH was determined using a multi-parameter water quality tester (HACH, Loveland, CO, United States), and TOC was determined using an elemental analyzer (Vario MACRO cube, Elementar, Germany). The amount of un-ionized NH_3_ based on pH was calculated using the following formula (Emerson et al., 1975):


$${NH}_{3}\left(\mathrm{mg}\cdot {\mathrm{kg}}^{-1}\right)=1/\left(10^{pKa-pH}+1\right)$$

Where p*K*_a_ is the dissociation constant of NH_3_ + H^+^/NH_4_^+^ pair in solution.

### 2.4 DNA extraction, sequencing and quantification of AOA and AOB

Total DNA was extracted from 0.5 g of freeze-dried weathered rocks and sediments using the FastDNA^®^ SPIN kits for soil (MP Biomedicals, United States). The concentration and quality of the extracted DNA were determined using a Nanodrop 2000 (ND2000, Thermo Fisher Scientific) spectrophotometer for subsequent experiments. AOA was amplified using the primer set of Arch-amoA26F/Arch-amoA417R (Park et al., 2008), and the primer set of amoA1F/amoA2R was used for AOB (Weiner and Maizels, 1999). Paired-end sequencing of the *amoA* functional genes of AOA and AOB was performed on the Illumina Miseq PE300 platform at Shanghai Personal Biotechnology, Co., Ltd., Shanghai, China. Quantification of the *amoA* genes of AOA and AOB was performed using the primer sets of Arch-amoAF/Arch-amoAR and amoA1F/amoA2R, respectively, with the systems and reactions as described previously (Rotthauwe et al., 1997; Francis et al., 2005; Gao et al., 2016). The *R*^2^ values of the standard curves were 0.95 or higher in this study. The abundance of each gene was normalized to the number of qPCR-derived gene copies per gram of dry weight sample. All raw sequence reads were deposited in National Omics Data Encyclopedia (NODE) with the project numbers OER444534 for AOA and OER445462 for AOB.

### 2.5 Sequence processing and bioinformatics analysis

Primer fragments were excised with *cutadapt* plugin, sequences were spliced with the fastq_mergepairs module of Vsearch, and quality control was performed with the fastq_filter module. Repetitive sequences and chimeras were removed with the derep_fulllength module and uchime_denovo module, respectively. A perl^1^ script was run to filter chimeras from the sequence set after quality control. High quality sequences were clustered at 85% similarity (Pester et al., 2012). Species annotation was performed using the National Center for Biotechnology Information (NCBI) database.^2^ The sequence numbers of *amoA* genes were, respectively, resampled to 58,405 (for AOA) and 39,470 (for AOB) reads to avoid the influence of sequencing depth on microbial diversity.

Alpha diversity indices (Chao 1, Shannon, Simpson), and principal coordinate analysis (PCoA) were calculated via the *vegan* and *ggplot* packages in R (Dixon, 2003). The permutational multivariate analysis of ANOVA (PERMANOVA) was conducted based on Bray–Curtis dissimilarity via the *vegan* package in R (Rui et al., 2015). Redundancy analysis (RDA) of environmental factors and relative abundance of dominant OTUs were conducted with Canoco 5, and co-occurrence network of ammonia-oxidizing microorganisms was analyzed with *Hmisc* package and were visualized with the Fruchterman-Reingold layout in Gephi (Bastian et al., 2009). Keystone taxa was characterized using betweenness centrality values (Jiao et al., 2016; Xiang et al., 2017). Community construction was based on iCAMP in the R package implemented in the Galaxy platform online website^3^ (Ning et al., 2020). The Pearson test was used in the correlation between βNTI and environmental factors for those that conformed to a normal distribution and the Spearman test for those that did not. The niche breadth index was calculated using the *spaa* package in R. The phylogenetic tree was constructed by selecting the dominant OTUs with >10% abundance and using the National Center for Biotechnology Information (NCBI) GenBank database for BLAST comparison to obtain highly homologous sequences (> 97%) (Liu H. Y. et al., 2023). Phylogenetic analysis was performed using the maximum likelihood method using MEGA 11 software (Ren et al., 2023).

### 2.6 Potential nitrification rate (PNR)

PNR measurements were conducted in triplicates with two experimental groups. The air-dried sample of 5 g was transferred into a 50 mL corning tube and 20 mL of phosphate buffer was added. 1 mM (NH_4_)_2_SO_4_ was added into the centrifuge tubes to serve as the substrate for ammonia oxidation (Zhao et al., 2017). The control groups were treated with 800 μg/mL kanamycin to inhibit the growth of AOB (Taylor et al., 2010). Subsequently samples were incubated at 19°C (close to the mean annual temperature in XCT Cave) for 24 h in the dark at rest followed by the addition of 1 mM KClO_3_ to stop nitrite oxidation (He et al., 2007; Tourna et al., 2011; Zhao et al., 2017). Nitrite concentration was measured with sulfonamide colorimetric method with a 1:4 ratio of sulfonamide to nitrite (Yu et al., 2007). Variation of nitrite was used to calculate the potential nitrification rate based on the formula of:


$${PNR}_{AOA}\left(\mu M\cdot g^{-1}\right)=C_{\mathrm{kanamycin}}{\mathrm{NO}}_{2}^{-}/5$$

$${PNR}_{AOB}\left(\mu M\cdot g^{-1}\right)=C_{\mathrm{no}-\mathrm{kanamycin}}{\mathrm{NO}}_{2}^{-}/5-{PNR}_{AOA}$$

The definition of abbreviation in the formula are showed as follow:

PNR_AOA_: the potential nitrification rate by AOA; C_kanamycin_NO_2_^−^: NO_2_^−^ concentration in the system with the addition of kanamycin; C_no-canamycin_NO_2_^−^: NO_2_^−^ concentration in the system without the addition of kanamycin; PNR_AOB_: potential nitrification rate of AOB.

## 3 Results

### 3.1 Changes of physicochemical parameters with niches

The NH_4_^+^ concentration was higher in the sediment samples than those in weathered rock. Toward the interior of the cave, NH_4_^+^ concentration decreased, and NH_3_ increased (Table 1). The highest NH_4_^+^ concentration (23.43 ± 4.54 mg/kg) was observed in the sediment collected in entrance nearby zone (ENZ-S), and the lowest NH_4_^+^ concentration (10.40 ± 4.15 mg/kg) was observed in the weathered rock collected in the dark inner zone (IZ-W). The variation of NH_3_ ranged from 88.04 ± 22.46 to 151.53 ± 47.29 mg/kg (Table 1). All samples were weakly alkaline and the pH value (8.03 ± 0.47 and 8.38 ± 0.40) of IZ samples was higher than those of ENZ samples (7.69 ± 0.47 and 7.81 ± 0.18) (Table 1). Temperature in inner zone was higher (16.30 ± 0.92°C) than that in ENZ (10.00 ± 0.14°C).

**Table 1:** Physicochemical parameters of weathered rocks and sediments within the XCT Cave, Guilin City, Guangxi Province.

Sample	ENZ-S	IZ-S	ENZ-W	IZ-W
pH	7.81 ± 0.18^a^	8.38 ± 0.40^a^	7.69 ± 0.19^a^	8.03 ± 0.47^a^
K^+^ (mg/kg)	2.50 ± 0.33^a^	1.88 ± 0.78^a^	3.60 ± 0.64^a^	44.73 ± 30.50^a^
TOC (%)	1.99 ± 0.04^a^	3.00 ± 1.67^a^	1.83 ± 1.02^a^	3.58 ± 0.70^a^
Cl^−^ (mg/kg)	1.65 ± 0.06 ^a^	1.10 ± 0.38 ^a^	2.44 ± 0.81 ^a^	12.80 ± 6.21^a^
NO_3_^−^ (mg/kg)	16.36 ± 11.57^a^	6.32 ± 2.40^a^	6.11 ± 3.42^a^	349.48 ± 268.28^a^
SO_4_^2−^ (mg/kg)	20.82 ± 3.02^a^	17.82 ± 2.26^a^	27.61 ± 4.69^a^	1,345 ± 1,147.66^a^
NH_4_^+^ (mg/kg)	23.43 ± 4.54^a^	16.41 ± 3.50^a^	13.28 ± 3.83^a^	10.40 ± 4.15^b^
Mg/Si	0.09 ± 0.07^a^	0.05 ± 0.06^a^	0.24 ± 0.17^a^	1.41 ± 1.06^a^
Ca/Si	4.10 ± 0.65^a^	4.37 ± 0.44^a^	6.46 ± 0.13^a^	32.77 ± 21.63^a^
Temperature (°C)	10.00 ± 0.14^b^	16.30 ± 0.92^a^	10.00 ± 0.14^b^	16.30 ± 0.92^a^
NH_3_ (mg/kg)	88.04 ± 22.46^a^	114.02 ± 53.70^a^	114.91 ± 39.58^a^	151.53 ± 47.29^a^

Different letters (a, b) show significant difference (*p* < 0.05) among groups based on one-way ANOVA. ENZ-S, sediments collected from the entrance nearby zone in the XCT Cave; ENZ-W, weathered rock samples collected from the entrance nearby zone; IZ-S, sediment collected from the inner zone; IZ-W, weathered rock collected from the inner zone.

### 3.2 Diversity and composition of ammonia-oxidizing microbial communities

**Figure 1:**
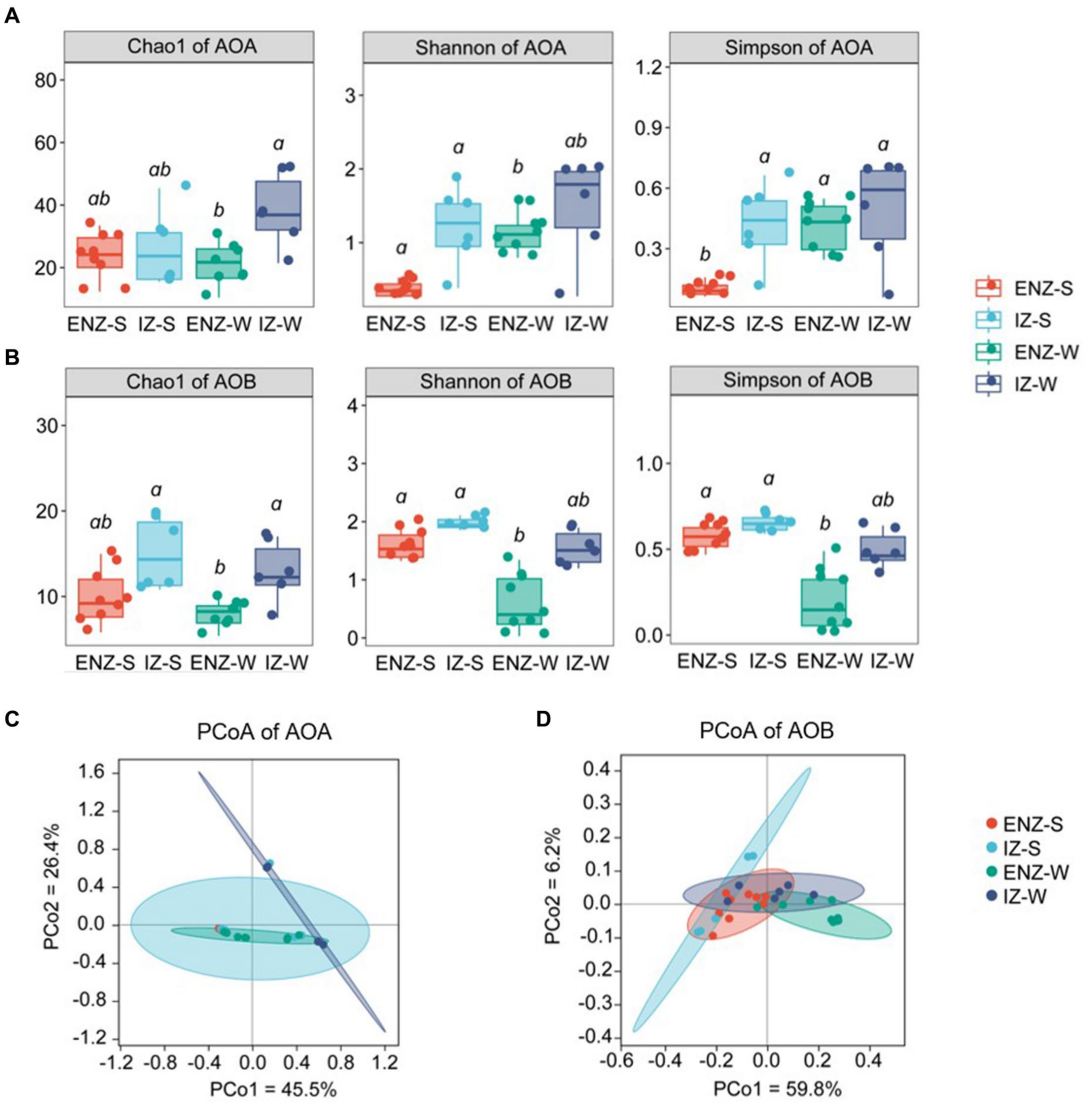
Alpha diversity of ammonia-oxidizing archaea **(A)** and ammonia-oxidizing bacteria **(B)** within the XCT Cave, Guilin City. Different letters (a, b) above the box show significant difference (*p* < 0.05) among groups based on one-way ANOVA. Panels **(C,D)** revealed beta diversity based on the Bray-Curtis distance based on *amoA* gene sequences of ammonia-oxidizing archaea and ammonia-oxidizing bacteria as indicated by PCoA plots, respectively. ENZ-S, sediments collected from the entrance nearby zone; ENZ-W, weathered rock samples collected from the entrance nearby zone; IZ-S, sediment from the interior zone; IZ-W, weathered rock from the interior zone.

Alpha diversity of ammonia-oxidizers was significantly different among different niches. Overall AOA communities showed higher alpha diversity than those of AOB as indicated by Chao 1 index, Simpson index and Shannon index (Figure 1). The AOA alpha diversity in inner zone was higher than that that in the entrance nearby zone, and the alpha diversity of the weathered rock was higher than that of the sediments (Figure 1A). In contrast, AOB showed a higher alpha index in IZ than those in ENZ, whereas the significant difference was only found in the rock samples in ENZ and sediments in IZ (Figure 1B). PCoA analysis showed significant differences in AOA (*p* = 0.002) and AOB (*p* = 0.001) communities between different niches such as sediments versus weathered rocks and samples in ENZ versus those in IZ (Figures 1C,D).

**Figure 2:**
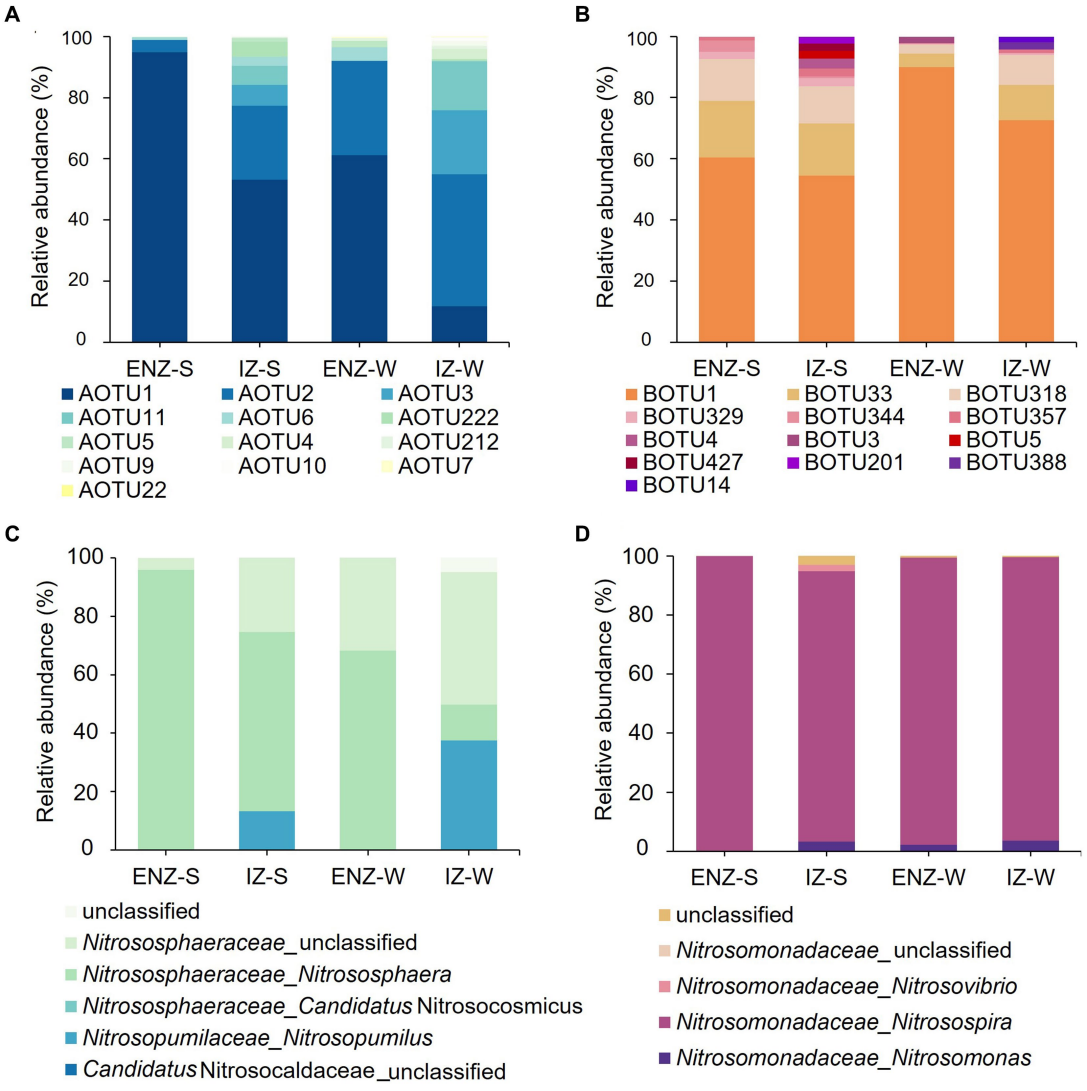
Compositions of ammonia oxidizing archaea and bacteria within the XCT Cave. The relative abundance of ammonia oxidizers at the OTU level (top 13) **(A)** and the genus level **(C)**. The compositions of ammonia-oxidizing bacteria at the OTU level (top13) **(B)** and the genus level **(D)** in the XCT Cave. Abbreviations of ENZ-S, ENZ-W, IZ-S ang IZ-W are the same as those in Figure 1.

A total of 200 AOA OTUs was recovered from the cave samples and OTU1 was numerically dominant in all habitats (Figure 2A). All the AOA OTUs were affiliated with *Thaumarchaeota*, which was furthered divided into two orders (*Nitrosopumilales* and *Nitrososphaeria*) and three genera (*Nitrososphaera*, *Candidatus* Nitrosocosmicus, and *Nitrosopumilus*). AOTU1 dominated AOA communities with the highest relative abundance of 94.86%, 53.17%, and 61.16% in ENZ-S, IZ-S and ENZ-W, respectively, while AOTU2 dominated IZ-W with the relative abundance of 43.17% (Figure 2A). At the genus level, *Nitrososphaera* was dominant in samples from ENZ-S, IZ-S and ENZ-W, accounting for 95.70%, 61.28%, and 68.18%, respectively. However, an unclassified genus of *Nitrosophaeraceae* had the highest relative abundance (45.30%) in IZ-W, followed by the *Nitrosopumilus* with a relative abundance of 37.54% (Figure 2C).

In total 35 AOB OTUs were assigned with three genera, *Nitrosomonas*, *Nitrosospira*, and *Nitrosovibrio*, which belonged to the orders of *Nitrosomonadales* and *Nitrosomonadaceae*, affiliated to *Betaproteobacteria* class. BOTU1 dominated in AOB communities with the relative abundance of 60.47%, 54.42%, 90.00%, and 72.54% in ENZ-S, IZ-S, ENZ-W and IZ-W, respectively (Figure 2B). Whereas, at the genus level, *Nitrosospira* dominated in all samples with 99.91%, 91.57%, 97.24%, and 95.94% in ENZ-S, IZ-S, ENZ-W, and IZ-W, respectively (Figure 2D).

**Figure 3:**
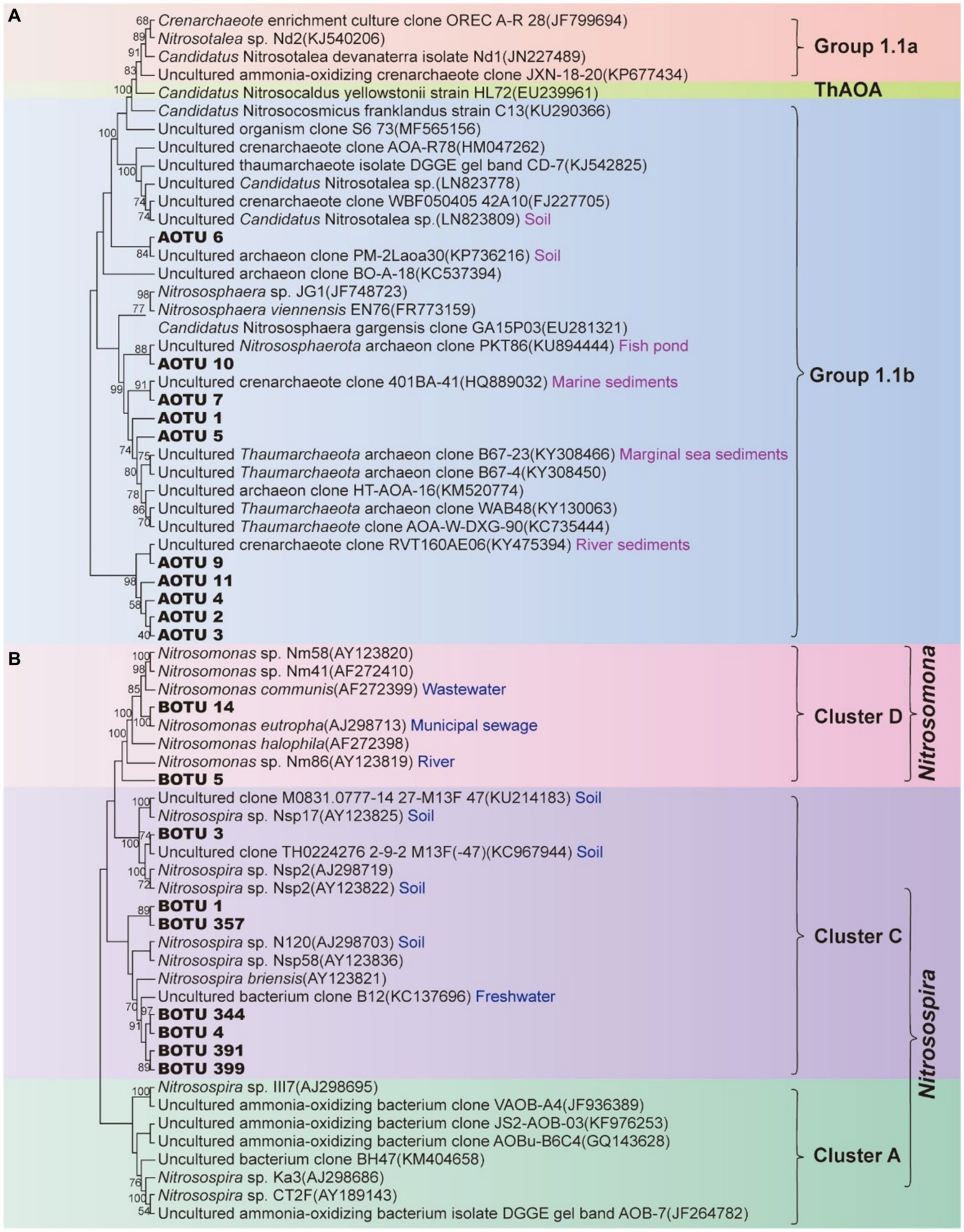
Phylogenetic tree of ammonia-oxidizing archaea **(A)** and ammonia-oxidizing bacteria **(B)** based on *amoA* gene sequences. Branching patterns in the maximum likelihood tree were expressed using the respective bootstrap values (1,000 iterations). OTU in bolds are from this study. AOTU, archaeal OTU; BOTU, bacterial OTU.

Phylogenetically all the dominant AOA OTUs (AOTU) in the XCT Cave belonged to Group I.1b (Figure 3A) and the AOB OTUs (BOTU) belonged to Cluster D and Cluster C (Figure 3B).

### 3.3 Absolute abundance of *amoA* gene, PNR, co-occurrence network and community assembly of ammonia-oxidizers

**Figure 4:**
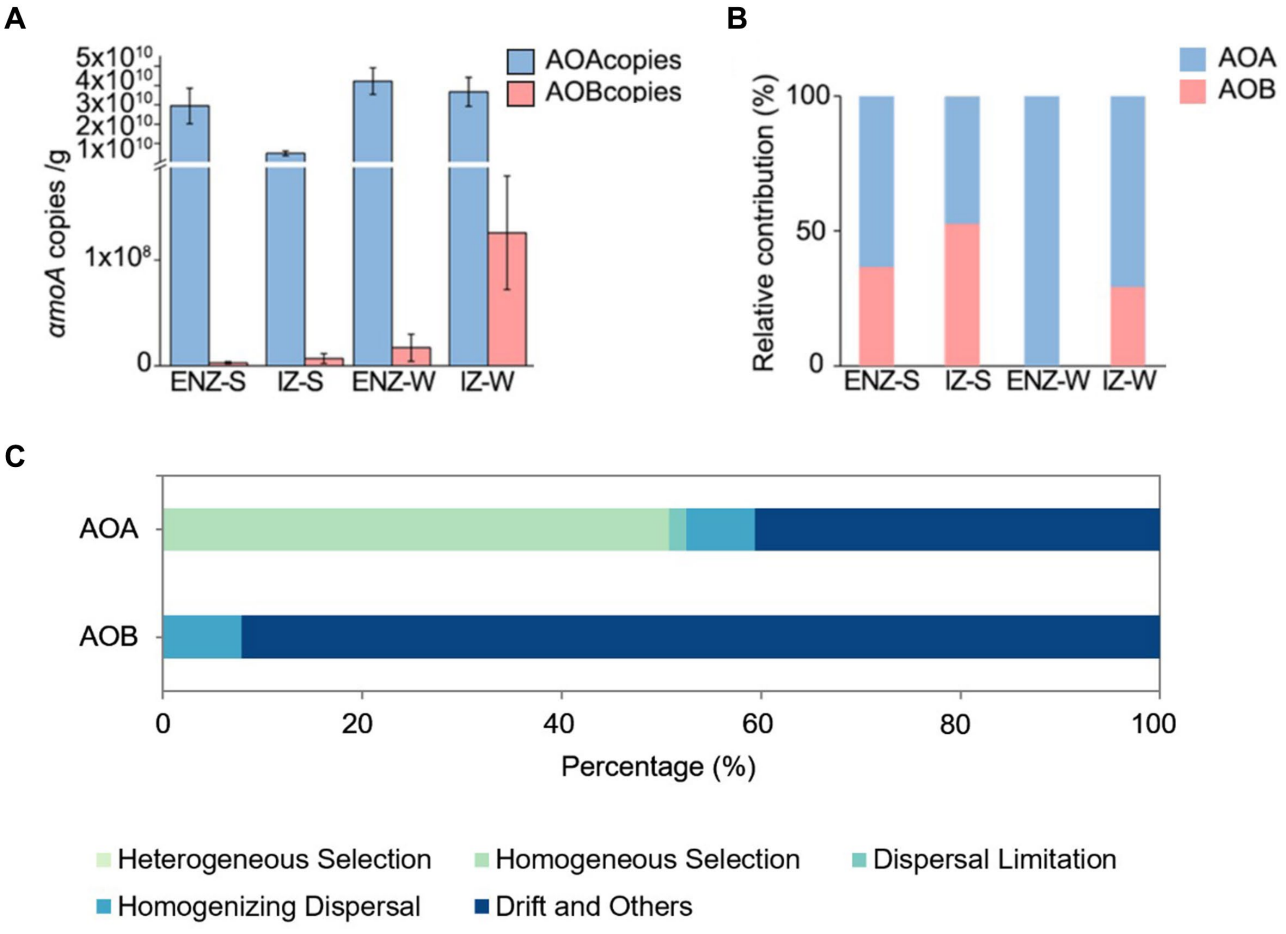
Absolute abundances of archaeal and bacterial *amoA* genes in the XCT Cave **(A)**. Relative contribution to nitrification by AOA and AOB in the XCT Cave **(B)**. The relative contribution of different ecological processes to ecological assembly processes of ammonia-oxidizing archaea and ammonia-oxidizing bacteria communities **(C)**. Abbreviations of ENZ-S, ENZ-W, IZ-S ang IZ-W are the same as those in Figure 1.

The AOA outnumbered AOB by 2 to 6 orders magnitude as confirmed by the quantification of *amoA* gene. In samples close to the cave entrance, the absolute abundance of AOA *amoA* gene ranged from 3.02 × 10^10^ to 6.25 × 10^10^ copies/g in weathered rock, whereas AOB was ranging from below detection to 4.87 × 10^7^ copies/g (Figure 4A). In sediments samples, AOA varied from 5.05 × 10^9^ to 6.64 × 10^10^ copies/g, whereas the AOB ranged from 1.76 × 10^5^ to 7.48 × 10^6^ copies/g (Figure 4A). In the inner zone, AOA had 1.73 × 10^10^ to 5.61 × 10^10^ copies/g in weathered rock, while AOB ranged from 2.48 × 10^4^ to 2.51 × 10^8^ copies/g sample. In sediments samples, AOA had 4.22 × 10^9^ to 5.62 × 10^9^ copies/g, and AOB varied from 1.78 × 10^4^ to 6.85 × 10^6^ copies/g (Figure 4A).

Weathered rock samples showed higher PNR (0.37 ± 0.24 μM/g) than those in the sediment samples (0.22 ± 0.11 μM/g) (Supplementary Table S1) and AOA contribute more to PNR than AOB within the XCT Cave (Figure 4B). AOA exclusively contributed to PNR in rock samples close to the entrance and AOB contributed 52% to PNR in the sediments in IZ (Figure 4B).

**Figure 5:**
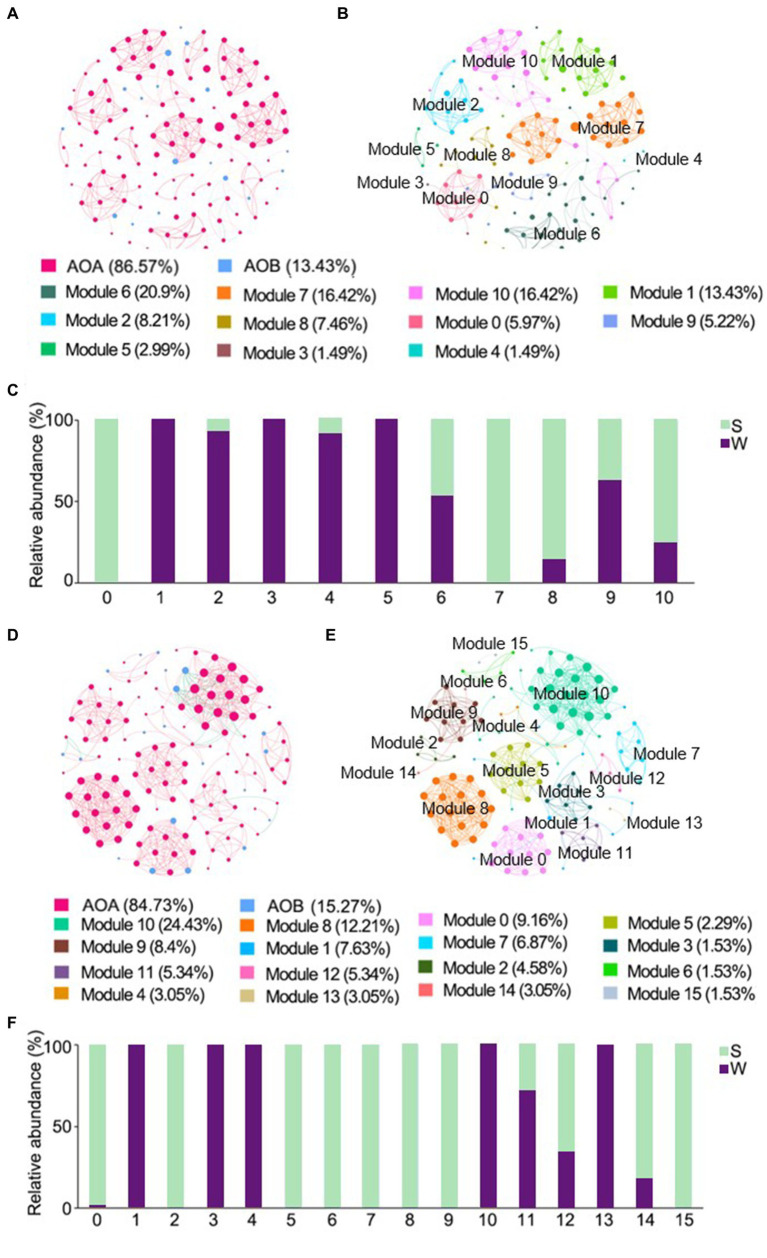
Cross-domain co-occurrence networks based on pairwise Spearman’s correlations between OTUs with a coefficient > |0.6| and a *p*-value <0.01. Cross-domain co-occurrence networks of ENZ with OTUs colored by ammonia-oxidizers **(A)** and modularity **(B)**, and the histogram represented the relative abundance of nodes in each module in weathered rocks and sediments **(C)**. Cross-domain co-occurrence networks of IZ with OTUs colored by ammonia-oxidizers **(D)** and modularity **(E)**, and the histogram represented the relative abundance of nodes in each module in weathered rocks and sediments **(F)**. The size of each node is proportional to the number of connections. Red lines represent positive correlations and green lines represent negative correlations. W, weathered rock; S, sediment.

The ENZ network of ammonia-oxidizers consisted of 134 nodes and 429 edges, and the IZ network consisted of 131 nodes and 546 edges, respectively (Table 2). Positive links dominated in all networks. Higher weighted degree, diameter, and modularity index were observed in the ENZ network compared with the IZ network, whereas the topology indices of density and mean clustering coefficient were higher in the IZ network (Table 2). The nodes of AOA predominated in all networks, accounted for 86.57% and 84.73% in the networks of ENZ (Figures 5A,B) and IZ (Figures 5D,E), respectively. More nodes were from weathered rocks in ENZ networks (56%) (Figure 5C), while more nodes were from sediments in the IZ networks (65%) (Figure 5F).

**Table 2:** Topology indices of ENZ and IZ co-occurrence network of ammonia oxidizing archaea and ammonia-oxidizing bacteria within the XCT cave, Guilin City, Guangxi Province.

Location	Nodes	Edges	Weighted degree	Diameter	Density	Modularity index	Mean clustering coefficient
ENZ	134	429	11.181	9	0.048	0.857	0.841
IZ	131	546	7.976	8	0.064	0.827	0.906

ENZ, the entrance nearby zone; IZ, the interior zone.

The identification of keystone taxa showed that the most dominant taxon in the ENZ network was OTU320 (*Nitrosopira*) with the highest betweenness centrality value of 221, followed by OTU3 (*Nitrosopumilus*) with the highest betweenness centrality value of 194, in addition to *Nitrososphaera*, *Nitrosomonas*, *Nitrosospira* and *Candidatus* Nitrosocosmicus (Table 3). In contrast, the keystone taxa in the IZ network were *Nitrosopira*, *Nitrososphaera*, and *Nitrosopumilus*, with the highest betweenness centrality values 130 of OTU344 (*Nitrosopira*) (Table 3).

**Table 3:** Betweenness centrality of the top 10 nodes in the co-occurrence network of ammonia-oxidizing archaea and ammonia-oxidizing bacteria in ENZ and IZ of the XCT Cave, Guilin City, Guangxi Province.

ENZ			IZ
Ammonia-oxidizing microbes	Genus	Betweenness centrality	Ammonia-oxidizing microbes	Genus	Betweenness centrality
AOB(OTU320)	*Nitrosospira*	221	AOB(OTU344)	*Nitrosospira*	130
AOA(OTU3)	*Nitrosopumilus*	194	AOB(OTU320)	*Nitrosospira*	108
AOA(OTU22)	*Nitrosopumilus*	140	AOB(OTU341)	*Nitrosospira*	84
AOA(OTU1)	*Nitrososphaera*	50	AOB(OTU329)	*Nitrosospira*	58
AOB(OTU329)	*Nitrosospira*	26	AOA(OTU204)	*Nitrososphaera*	30
AOB(OTU3)	*Nitrosomonas*	22	AOA(OTU6)	*Nitrososphaera*	30
AOA(OTU211)	*Candidatus* Nitrosocosmicus	16	AOA(OTU30)	*Nitrosopumilus*	28
AOA(OTU5)	*Nitrososphaera*	15	AOA(OTU120)	*Nitrososphaera*	24
AOB(OTU360)	*Nitrosospira*	11	AOA(OTU3)	*Nitrosopumilus*	14
AOA(OTU72)	*Nitrososphaera*	9	AOA(OTU22)	*Nitrososphaera*	13

ENZ, the entrance nearby zone; IZ, the interior zone.

Deterministic process dominated in AOA community assembly with a contribution of 50.71% (especially, homogeneous selection) (Figure 4C). NO_3_^−^, NH_4_^+^, TOC, and Cl^−^ were the environmental factors that significantly affected the βNTI of AOA (Table 4). Contrasting with the ecological processes for AOA community assembly, stochastic processes contributed 100% to AOB community assembly, dominated by drift with a contribution of 92.09% (Figure 4C). None of the environmental factors investigated in this study had a significant correlation on the βNTI of AOB (Table 4).

**Table 4:** Pearson and Spearman correlation analysis of environmental variables with βNTI of AOA and AOB in the XCT Cave, Guangxi Province.

Environmental variables	βNTI_AOA_	βNTI_AOB_
pH	−0.036	0.052
K^+^	−0.199	0.342
NO_3_^−^	−0.687^*^	0.023
NH_4_^+^	−0.646^*^	−0.001
TOC	−0.891^**^	−0.101
Cl^−^	−0.685^*^	−0.035
SO_4_^2−^	−0.273	−0.166
Mg/Si	−0.322	−0.264
Ca/Si	−0.6	0.345
Temperature	−0.283	0.123

*p* < 0.05 was considered significantly different and indicated by *. ^*^p < 0.05 and ^**^p < 0.01.

### 3.4 Correlation between environmental factors and ammonia-oxidizers and their niche breadth

**Figure 6:**
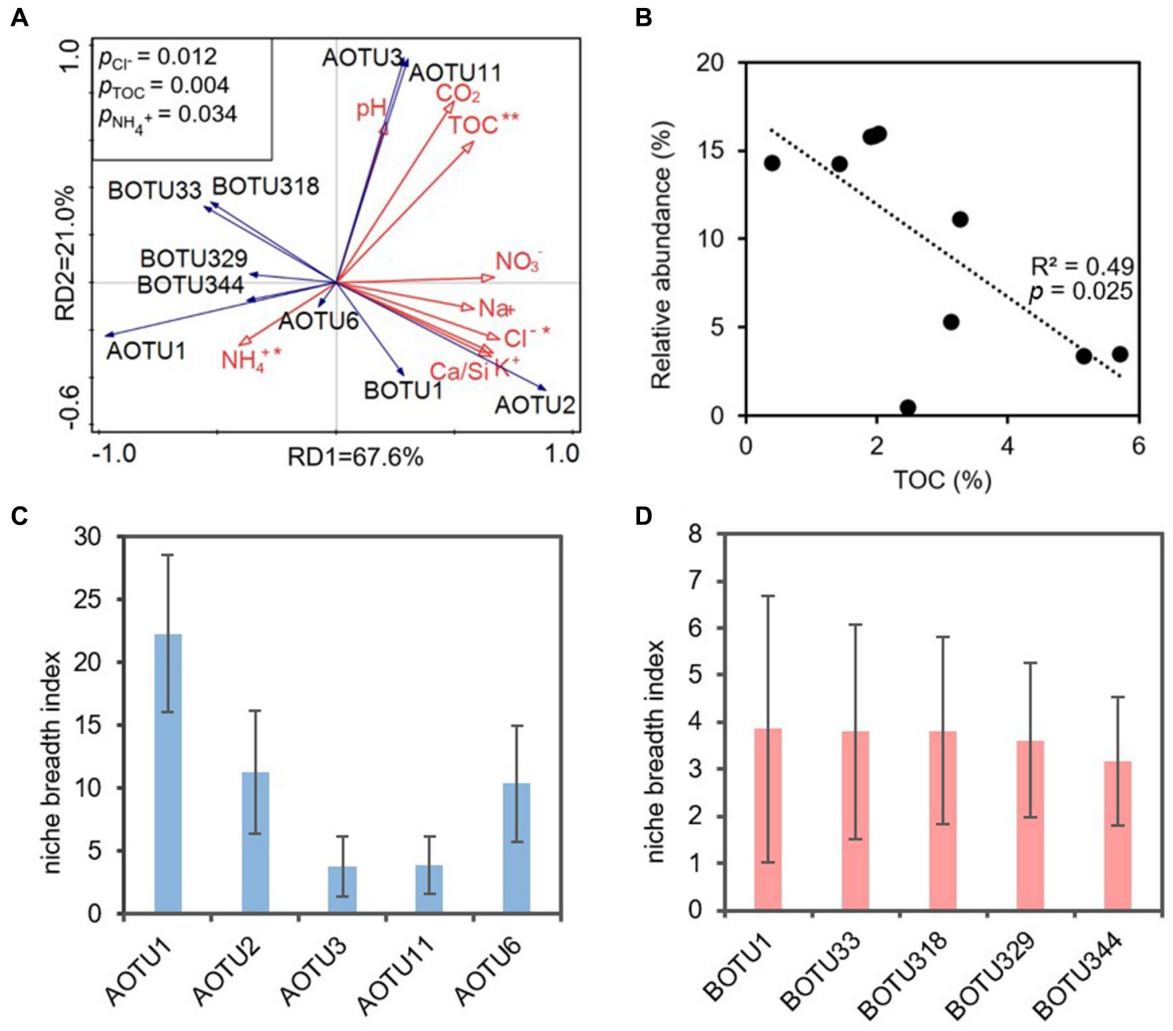
**(A)** Redundancy analysis (RDA) of physicochemical parameters (red solid arrows) and dominant OTUs (top 5) (blue solid arrows) based on ammonia-oxidizing archaeal and ammonia-oxidizing bacterial *amoA* genes. AOTU, archaeal OTU; BOTU, bacterial OTU. Asterisks indicate statistical significance (^*^p < 0.05, ^**^p < 0.01, and ^***^p < 0.001), and the linear fit of TOC to the AOTU1 relative abundance **(B)**. Panel **(C,D)** revealed the niche breath index of the dominant OTUs of AOA and AOB, respectively.

The RDA analysis indicated that TOC, Cl^−^ and NH_4_^+^ significantly impacted on ammonia-oxidizers (*p* < 0.05). RD1 and RD2 explained 67.60% and 21.00% of the variance, respectively (Figure 6A). Among them, TOC was positively correlated with AOTU3, AOTU11, AOTU2, and negatively correlated with most AOB OTUs and AOTU1, AOTU6 (Figure 6A). A linear fit of TOC to the AOTU1 showed that a negative correlation between the content of TOC and the relative abundance of AOTU1, *R*^2^ = 0.49 (Figure 6B). However, the linear fit effect of to the OTU of AOB was poor (Supplementary Figure S1).

The niche breadth index showed that the dominant OTUs of AOA showed much wider niche breadth compared to that of AOB. The niche breadth index of AOA ranged from 3.8 (AOTU1) to 22.3 (AOTU3) (Figure 6C), whereas the BOTUs had niche breadth index ranging from 3.2 to 3.9 (Figure 6D).

## 4 Discussion

### 4.1 Better adaption of AOA in the oligotrophic karst cave than their bacterial counterpart

The niche breadth index provided robust support for the superior adaption of AOA in oligotrophic karst cave compared to their bacterial counterparts. The elevated niche breadth index (Figures 6C,D) signified that AOA might effectively exploit a broader spectrum of resources in oligotrophic conditions when contrasted with AOB. On the contrary, the narrower niche breadth index indicated that AOB encountered environmental stress and heightened competition for resources (Vorobeichik, 1993; Martorell et al., 2015; Pannek et al., 2016).

The low concentration of ammonium in our cave strongly supports the dominance of AOA. AOA outnumbered their ammonia-oxidizing counterpart AOB by up to thousands of times in the copy numbers of *amoA* gene within the XCT Cave (Figure 4A), and contributed over 50% to PNR (Figure 4B), which matched well with the observation in other oligotrophic environments (Zhao et al., 2017; Marques et al., 2018; Cardarelli et al., 2020). Usually, AOA show high affinity to ammonium thus achieves a competitive advantage under oligotrophic conditions (Martens-Habbena et al., 2009). Experimental evidence with enriched culture of AOA and AOB from freshwater environments supported this, revealing AOA dominance at an initial ammonium concentration of 50 μM, while AOB dominate at 500 μM (French et al., 2021). A moderately thermophilic ammonia-oxidizing archaea is partially inhibited by a concentration of 3.08 mM ammonium, whereas active at 0.14 and 0.79 mM ammonium (Hatzenpichler et al., 2008). The ammonium concentrations in our cave varied from 10.40 ± 4.15 to 23.43 ± 4.54 mg/kg, equivalent to 0.58 ± 0.23 to 1.30 ± 0.25 mM (Table 1), favored for the establishment and thriving of AOA communities.

pH is another fundamental factor selecting on the nitrifying communities in natural environments (Li et al., 2018; Aigle et al., 2019). *Thaumarchaeota* Groups I.1a and I.1b are generally associated with neutrophilic or alkaline limestone systems (Chelius and Moore, 2004; Spear et al., 2007; Tetu et al., 2013), whereas *Thaumarchaeota* Group I.1c tend to dominate in sandstone caves with the pH ranging from 3 to 7 (Barton, 2013; Zhao et al., 2017; Marques et al., 2018). All of our dominant AOTUs affiliated with Group I.1b (Figure 3A), indicating a carbonate cave niche specificity. Compared with Group I.1a, the higher NH_3_ affinity [*K*_m(app)_ ≈ 0.14–31.5 μM] (Jung et al., 2022) may help Group I.1b to dominate in karst caves. Due to the ionization of ammonia to ammonium, ammonia concentration decreases exponentially with the decreasing pH (Allison and Prosser, 1993; Wang et al., 2019). Therefore, AOA contributes more to nitrification under acidic pH conditions (Li et al., 2018, 2019; He et al., 2020). Different from the observation of the dominance of AOA in acidic soils, AOA has also been confirmed to dominate in alkaline and oligotrophic cave sediments (Zhao et al., 2017), which matched our results in this study (Figures 4A,B). At alkaline conditions with a pH > 7.3, a significant portion of the NH_3_/NH_4_^+^ pair exists as NH_3_, leading to NH_3_ concentrations exceeding 1 mM in copiotrophic systems. This creates an environment where AOB become more competitive. However, based on the p*K*_a_ of ammonia, it’s worth noting that 1 mM NH_3_ is still 100-fold lower than the ammonia concentration typically used for AOB culturing (1.2 mM NH_4_^+^ equivalent to 100 mM NH_3_) (Martens-Habbena et al., 2009; Pester et al., 2011). The NH_3_ concentration in our cave, determined by the ionization constant of NH_3_ to NH_4_^+^, ranged from 5.18 ± 1.32 to 8.91 ± 2.78 mM. Although this range still favored the dominance of AOA, there was potential for competition from AOB for nitrogen resource to some extent.

The PCoA analysis indicated a pronounced segregation in AOA communities compared to AOB (Figures 1C,D), suggesting robust environmental selection on AOA (D'Amen et al., 2018; Shen et al., 2022). This aligned with the dominance of homogeneous selection in AOA community assembly (Figure 4C), revealing a strong influence of environmental factors driving AOA communities towards convergence (Wu et al., 2021). Pearson and Spearman tests furthered confirmed the significant impact of NO_3_^−^, NH_4_^+^, TOC and Cl^−^ on AOA community assembly in our study (Table 4). In line with our findings, an increase in NH_4_^+^ supply has been reported to shift the ecological process from deterministic to stochastic process in AOA community assembly (Ma et al., 2023). This implied that AOA communities exhibited greater competitiveness and adaptability to oligotrophic conditions.

In contrast, AOB community assembly exhibited a predominance stochastic processes (Figure 4C), suggesting that the development of AOB communities was more self-regulated by intrinsic factors (Zhou and Ning, 2017). This observation was further supported by the Pearson and Spearman tests, revealing no significant correlations between environmental factors and βNTI of AOB (Table 4). Similar patterns have been demonstrated under other oligotrophic conditions, where AOB community establishment is also dominated by stochastic process (Yang et al., 2022; Fang J. et al., 2023). The dominance of genetic drift in the development of the AOB community in our study (Figure 4C) may suggest a relatively small AOB community (Ma et al., 2023; Ye et al., 2023; Zhang et al., 2023). This could be attributed to the challenges that AOB face in colonizing under lower ammonium concentrations (Keerio et al., 2020).

### 4.2 Impacts of environmental variables and interaction between ammonia-oxidizers within the cave

TOC, NH_4_^+^ and Cl^−^ emerged as the primary environmental impact factors shaping ammonia oxidizers (Figure 6A). *Nitrososphaera* of AOA exhibited the dominance in the nearby zone of the cave entrance, declining in abundance inward to the cave, and was replaced by *Nitrosopumilus* in the IZ-W (Figure 2C). Similarly, IZ-W showed higher alpha diversity compared with other niches (Figure 1A). These findings suggest that environmental factors can influence the community composition and diversity of the AOA (Jin et al., 2011; Patil et al., 2021; Kou et al., 2023). The most abundant AOTU1 (Figures 2A) showed a negative correlation with TOC (Figure 6B), while the dominant OTU of AOB exhibited a positive correlation with TOC (Supplementary Figure S1). This aligned with findings in other environments, such as ponds, estuaries and upland soils (Dai et al., 2015, 2018; Wei et al., 2021; Zhu et al., 2023). Most AOA are autotrophs and well adapted to oligotrophic environments due to their highly efficient CO_2_-fixation pathway of hydroxypropionate/hydroxybutyrate (HP/HB) cycle, as indicated by genomic studies (Kobayashi et al., 2018; Zhao and Zhang, 2022). This may well explain the observation of the dominance of AOA in oligotrophic conditions and their higher contribution to nitrification in karst caves (Wang et al., 2021). However, lower TOC does not favor heterotrophic AOB in term of organic carbon sources. AOB typically show positive correlation with TOC (Zhang et al., 2010; Dai et al., 2018; Zhu et al., 2023).

Cl^−^ showed a negative correlation with the dominant OTUs of both AOA and AOB, indicating the inhibition of Cl^−^ on the functioning of *amoA* enzymes in ammonia-oxidizing microorganisms (Wang et al., 2014; Roy et al., 2020). Similar negative correlations have also been observed between Cl^−^ and ammonia oxidizers in drinking water (Scott et al., 2015) and demonstrate inhibition on nitrification (Roy et al., 2020).

The AOA also dominated in the co-occurrence networks with AOB in caves. AOA occupied a greater nodes number in the cross-domain networks, forming closer links inward to the cave (Figures 5C,F), which suggested more collaboration for enhanced survival in subsurface caves. Interestingly, in the ENZ network, most nodes belonged to the weathered rock samples (Figure 5C), whereas the IZ network exhibited more sediment nodes (Figure 5F). In some oligotrophic environments, AOA have been found to dominate the nitrification network despite of the higher number of AOB (Zheng et al., 2022; Hu et al., 2023; Liu J. J. et al., 2023). These finding collectively highlighted the crucial role of AOA in the stabilizing the network (Jones and Hallin, 2019; He et al., 2021; Ma et al., 2023).

*Nitrosospira* and *Nitrosopumilus* were the keystone taxa in the ENZ network, while *Nitrosospira* and *Nitrososphaera* were the keystone taxa in IZ (Table 3). *Nitrosospira* are widely distributed in terrestrial and marine ecosystems (Lebedeva et al., 1997; Jiang and Bakken, 1999; Hollibaugh et al., 2002). AOA isolates such as *Nitrosopumilus*, *Nitrososphaera*, *Nitrosopumilus,* and *Nitrososphaera* are characterized by small size, and harbor flagellum-encoding genes (Tourna et al., 2011; Stieglmeier et al., 2014; Qin et al., 2017; Bayer et al., 2019). Although AOA and AOB both survive in oligotrophic environments (Ming et al., 2020; Guo et al., 2022), *Nitrososphaera* is more commonly found in areas with arsenic contamination and high salinity compared to *Nitrosopumilus*. This suggests that *Nitrososphaera* exhibits higher resistance to extreme conditions (Li et al., 2013, 2014; Liu et al., 2018; Zhu et al., 2022).

## 5 Conclusion

This study provides compelling evidence showcasing the superior adaption of ammonia-oxidizing archaea to oligotrophic subsurface karst caves. Dominant AOA OTUs exhibited broader ecological niche indexes compared to dominant AOB OTUs. AOA dominated ammonia-oxidizing communities with higher *amoA* gene copy numbers and significant contribution (48–100%) to potential nitrification rate. Deterministic processes dominate the ecological processes for the establishment of AOA communities, and TOC and NH_4_^+^ were identified as the primary environmental influence on AOA community assembly. In contrast, AOB is mainly governed by stochastic processes. Ammonia-oxidizing archaea contributed more nodes in the co-occurrence networks with ammonia-oxidizing bacteria and they collaborated more with AOB to survive the extreme conditions. These findings deepen our understanding of the ecology of ammonia-oxidizing microorganisms and nitrogen cycles in the subsurface biosphere.

## Data availability statement

The datasets presented in this study can be found in online repositories. The names of the repository/repositories and accession number(s) can be found at: https://www.biosino.org/node/run/detail/OER444534, OER444534 for AOA and https://www.biosino.org/node/run/detail/OER445462, OER445462 for AOB.

## Author contributions

QL: Data curation, Formal analysis, Investigation, Methodology, Writing – original draft, Validation. XC: Data curation, Formal analysis, Investigation, Writing – review & editing. XL: Formal analysis, Investigation, Writing – review & editing. PG: Data curation, Validation, Writing – review & editing. HW: Conceptualization, Funding acquisition, Investigation, Project administration, Resources, Supervision, Writing – review & editing. CS: Investigation, Resources, Writing – review & editing. QH: Investigation, Writing – review & editing.

## Footnotes

^1^ https://github.com/torognes/vsearch/wiki/VSEARCH-pipeline

^2^ https://www.ncbi.nlm.nih.gov/guide/data-software/

^3^ http://ieg3.rccc.ou.edu:8080/
