# Supplementary material for: Ammonia-oxidizing archaea adapted better to the dark, alkaline oligotrophic karst cave than their bacterial counterparts
Source: Front Microbiol. 2024 Apr 10;15:1377721. doi: 10.3389/fmicb.2024.1377721 (PMC11041041; doi:10.3389/fmicb.2024.1377721)
Supplement: Supplementary file 1 [file Data_Sheet_1.pdf]

Ammonia-oxidizing archaea adapted better to the dark, alkaline oligotrophic karst cave than their bacterial counterparts

Qing Li [a, #], Xiaoyu Cheng [b, #], Xiaoyan Liu [a], Pengfei Gao [a], Hongmei Wang [a,b*], Chuntian Su [c], Qibo Huang [c]

[a]*School of Environmental Studies, China University of Geosciences, Wuhan, 430074, China*

[b] *State Key Laboratory of Biogeology and Environmental Geology, China University of Geosciences, Wuhan 430078, China*

[c] *Institute of Karst Geology, CAGS/Key Laboratory of Karst Dynamics, MNR & GZAR, Guilin, Guangxi 541004, China*

#These authors contribute equally to the work.

**Correspondence should be addressed to wanghmei04@163.com or hmwang@cug.edu.cn*

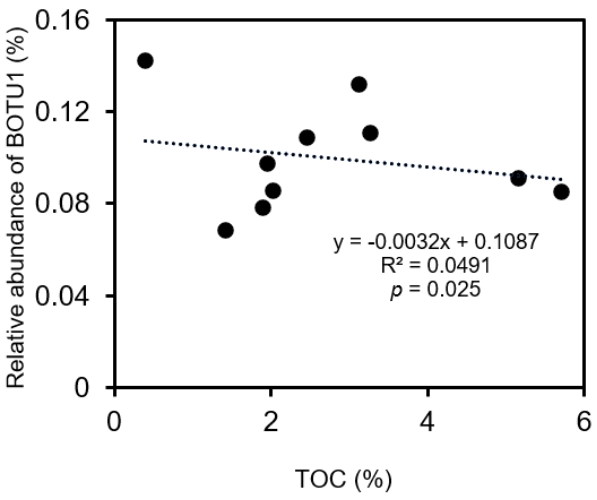

Fig. S1 Linear fit of TOC to the relative abundance of BOTU1.

Table S1. Sample location information and sequencing information in XCT Cave, Guangxi Province.

Sample ID	Location	Number of sequences (AOA)	Number of sequences (AOB)	Number of OTUs (AOA)	Number of OTUs (AOB)
S1_1	ENZ-S	78654	95253	18	6
S1_2	ENZ-S	87452	76264	13	7
S1_3	ENZ-S	91991	87132	13	9
S2_1	ENZ-S	86959	69428	20	7
S2_2	ENZ-S	110952	99801	18	7
S2_3	ENZ-S	101871	67190	17	12
S3_1	IZ-S	114365	58710	19	11
S3_2	IZ-S	94715	81874	24	9
S3_3	IZ-S	111498	75055	19	14
S4_1	IZ-S	87296	71695	13	11
S4_2	IZ-S	110398	73001	15	9
S4_3	IZ-S	117253	62499	15	11
S5_1	IZ-S	61479	48924	41	17
S5_2	IZ-S	74312	51184	29	16
S5_3	IZ-S	77722	57261	24	15
W1_1	ENZ-W	100473	69906	15	6
W1_2	ENZ-W	105435	81404	18	9
W1_3	ENZ-W	117704	88974	26	9
W2_1	ENZ-W	98393	97209	18	8
W2_2	ENZ-W	98228	82722	17	7
W2_3	ENZ-W	92115	83500	14	7
W3_1	IZ-W	111679	43273	14	7
W3_2	IZ-W	117128	80454	10	8
W3_3	IZ-W	118095	41548	19	6
W4_1	IZ-W	101852	66190	28	12
W4_2	IZ-W	113059	56223	21	12
W4_3	IZ-W	103712	99241	24	15
W5_1	IZ-W	107515	80358	45	12
W5_2	IZ-W	116335	76255	38	7
W5_3	IZ-W	109592	83750	35	17

S1_1: Biological parallel 1 of sediment at site1 in XCT Cave; W1_1: Biological paralled1 of weathered rock at site1 in XCT Cave.

Table S2. Potential nitrification rates of collected samples in XCT Cave, Guangxi Province.

Sample	Potential Nitrification Rate (μM g^{-1})	
	+	-
X1W	0.37 ± 0.02	0.22 ± 0.02
X2W	0.50 ± 0.13	0.39 ± 0.16
X3W	0.08 ± 0.05	0 ±0.00
X4W	0.74 ± 0.11	0.39 ±0.12
X5W	0.15 ± 0.03	0.11 ±0.14
X1S	0.17 ± 0.02	0.15 ±0.01
X2S	0.35 ± 0.04	0.20 ±0.06
X3S	0.35 ± 0.02	0.33 ±0.01
X4S	0.18 ± 0.09	0.13 ± 0.01
X5S	0.07 ± 0.01	0.02 ± 0.02

X1W: Weathered rock at site1 in XCT Cave; X1S: Sediment at site1 in XCT Cave; +: with kanamycin; -: without kanamycin.
